# The de Morton Mobility Index: Normative Data for a Clinically Useful Mobility Instrument

**DOI:** 10.1155/2012/353252

**Published:** 2012-09-04

**Authors:** E. M. Macri, J. A. Lewis, K. M. Khan, M. C. Ashe, N. A. de Morton

**Affiliations:** ^1^Centre for Hip Health and Mobility, University of British Columbia, Vancouver, BC, Canada V5Z 1M9; ^2^Department of Physiotherapy, Monash University, Melbourne, VIC 3800, Australia; ^3^Department of Family Practice, University of British Columbia, Vancouver, BC, Canada V6T 1Z4

## Abstract

Determining mobility status is an important component of any health assessment for older adults. In order for a mobility measure to be relevant and meaningful, normative data are required for comparison to a healthy reference population. The DEMMI is the first mobility instrument to measure mobility across the spectrum from bed bound to functional levels of independent mobility. In this cross-sectional observational study, normative data were obtained for the DEMMI from a population of 183 healthy, community-dwelling adults age 60+ who resided in Vancouver, Canada and Melbourne, Australia. Older age categories had significantly lower DEMMI mobility mean scores (*P* < 0.05), as did individuals who walked with a mobility aid or lived in semi-independent living (assisted living or retirement village), whereas DEMMI scores did not differ by sex (*P* = 0.49) or reported falls history (*P* = 0.21). Normative data for the DEMMI mobility instrument provides vital reference scores to facilitate its use across the mobility spectrum in clinical, research, and policymaking settings.

## 1. Introduction

The International Classification of Functioning (ICF) defines mobility as “moving by changing body position or location or by transferring from one place to another, by carrying, moving or manipulating objects, by walking, running or climbing, and by using various forms of transportation” [1]. Mobility is an important marker and predictor of physical abilities, independence, morbidity, and mortality [2–7]. Loss of mobility can result in a decline in independence, rendering individuals reliant on caregivers to meet their basic needs, or being unable to remain living independently. Such functional decline can also lead to injury and increased hospital admissions [8].

Determining mobility status is an important component of any medical or health assessment for older adults, whether an individual is acutely ill, living with chronic comorbidity, or is a healthy community dweller. Accurately measuring mobility can help a clinician determine an individual's abilities at a single time point to identify potential impairment or to establish a baseline for comparison following a subsequent reassessment. Mobility measures can also (i) help determine whether an individual's mobility has changed, (ii) identify early signs of decline, and (iii) assist with guiding therapeutic intervention, goal setting, and discharge planning in both inpatient and outpatient programs. Given the diverse functional abilities and complex health status of older adults, an ideal measure of mobility is clinimetrically sound, robust, and spans the spectrum of functionally relevant mobility tasks. Recent systematic reviews have revealed a plethora of mobility instruments for both acute medical patients and healthy community dwellers [9, 10]. Most of these instruments have many limitations, including the tendency to be designed for narrowly defined populations, resulting in floor or ceiling effects; lack of external validation or reliability testing; and/or demonstrated lack of responsiveness to change [9, 10].

The de Morton Mobility Index (DEMMI) was rigorously developed in response to clinical needs and has been previously shown to be a valid, reliable, and robust mobility instrument that can be used across the spectrum of mobility and across clinical settings [11–15]. It is easy to use, requires minimal equipment, and can be administered in a very short period of time, making uptake of this instrument into clinical practice both simple and attractive [11]. An important next step for improving usability of this instrument is the development of reference intervals (or “normative data”). Normative data regarding mobility are important for clinicians, researchers, and policy makers. Applied to individuals, they provide a comparison of the expected mobility levels for sex- and age-matched community-dwelling peers. Normative data can also provide richness to interpretation of population data, offering guidelines to help identify at risk populations or shape public health policy; it provides information that critically enhances interpretability.

Just as reference values are critical for the usefulness of measuring blood pressure, having accurate benchmarks for detecting mobility changes is essential for treatment, goal setting, and can serve to motivate both patients and clinicians. Given that normative data have such important, meaningful, and broad applications, the purpose of this study was to assess the mobility of community-dwelling older adults using the DEMMI and to develop reference intervals for healthy men and women over 60 years old.

## 2. Methods

### 2.1. Study Design

This was a cross-sectional observational study.

### 2.2. Participants

The study population comprised community-dwelling adults aged 60 years and older in large cities from two countries: Vancouver, Canada; Melbourne, Australia. We defined community dwelling as living in a house, apartment, or assisted living (AL)/retirement village. This decision was based on current definitions of AL in British Columbia, Canada [16] and retirement village in Victoria, Australia [17]. In both regions, this form of residence provides accommodation and some services that are distinct from the services provided in residential care/nursing home environments, and requires that residents are able to make decisions and otherwise live independently. An alternative definition of “community dwelling” includes those living in a house or apartment, but not AL (because AL is often described as “semi-independent”). We therefore presented our data both as a complete sample (i.e., using our working definition of community dwelling, provided remaining inclusion criteria were met); further separated our data into “independent” (defined as living independently in a house or apartment) and “semi-independent” (defined as living in AL or a retirement village), thus satisfying both definitions of “community dwelling” and achieving broader applicability.

Prior to participation, interested individuals were screened for eligibility to ensure they had no clinical conditions that might affect their mobility, including neuromuscular, orthopaedic, or cardiovascular impairments. All participants were required to speak English and were screened for cognitive limitations that would preclude the provision of informed consent. Where the ability to read was limited, the consent form was read aloud for these participants. All participants provided informed written consent. This study was approved for Canadian data collection by the Clinical Research Ethics Board at the University of British Columbia and by Vancouver Coastal Health Authority. The Australian portion of the study was approved by the Monash University Human Research Ethics Committee.

### 2.3. Recruitment

In Canada, recruitment strategies for this convenience sample included advertisements in local newspapers and on bulletin boards in seniors' centres, as well as word-of-mouth advertising by local fitness instructors. In Australia, a retirement village and Returned and Services League (RSL) members were invited to participate. Fliers were distributed to residents of the retirement village. Interested individuals contacted researchers by telephone to arrange an appointment for screening and participation or signed up at a specific site on the day of the assessments.

### 2.4. Data Collection

In Vancouver, Canada, assessments took place at eight different sites, including one AL residence, community centres, seniors' activity centres, and onsite at the Centre for Hip Health and Mobility, University of British Columbia. In Melbourne, Australia, assessments took place at two sites, one retirement village and one RSL centre. Each assessment lasted between 40–60 minutes. For the remainder of the paper we will use the term assisted living (AL) to include “retirement village”, since the terminology is simply a matter of dialect, and the two terms are virtually synonymous.

The primary outcome of interest, the DEMMI, is a performance-based instrument that assesses mobility through 15 hierarchical items that begin with bed mobility and progress through chair tasks, static balance, gait, and finally dynamic balance tasks. A raw ordinal score is converted (through Rasch analysis) to an interval score out of 100, with a higher score representing greater mobility. The DEMMI has been described in detail elsewhere [11, 18] and copies of the instrument are available in the original DEMMI publication [11] and at http://www.demmi.org.au/.

Demographic information gathered comprised age, sex, use of mobility aids, living situation, and medical comorbidities/history.

Research assistants or physiotherapists screened interested individuals for eligibility, obtained written informed consent, and conducted interviews, and administered the questionnaires. A physiotherapist conducted the DEMMI in Vancouver; in Melbourne, the DEMMI was administered by either a physiotherapist or a physiotherapy undergraduate honors student. The DEMMI was demonstrated by the test developer (NAD) to all persons who administered the test. A 30-minute instructional DVD (video available through http://www.demmi.org.au/) was also provided prior to data collection in Vancouver. The DEMMI instrument developer (NAD) was present during data collection in Australia and Canada to ensure procedural consistency.

### 2.5. Statistical Analysis

Exploratory data analysis included graphical exploration of the data, with descriptive statistics presented in tables. The DEMMI scores were explored by age category (60–69, 70–79, 80–89, and 90+), sex, and other categories such as living situation and use of mobility aid (e.g., cane, walker). The potential for a ceiling effect was investigated by calculating the percentage of participants achieving the maximum possible score of 100.

For normative data, reference intervals were constructed using empirical centiles (5th, 50th, and 95th) for the purposes of individual comparisons [19]; means and 95% confidence intervals were presented for purposes of population comparisons.

Visual inspection of univariate data as well as a scatter plot of the DEMMI by age and sex with overlying LOWESS (locally weighted scatterplot smoothing) was used to guide analysis. Welch's two-sampled *t*-tests were used to compare groups to account for heteroscedasticity. All statistical analysis was done using Stata Intercooled 12.0 (StataCorp, Texas, USA).

## 3. Results

**Figure 1:**
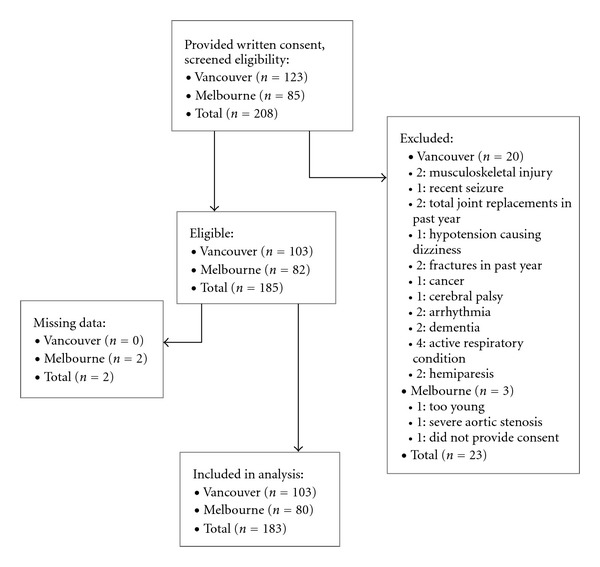
Flow diagram.

Initially, 208 individuals were screened for eligibility, and 23 were excluded, leaving 185 participants who completed the assessment (see Figure 1 for flow chart). Two participants had missing data for age and were therefore excluded from the analysis (see Figure 1).

A description of the 183 participants included in the analysis, including scores on questionnaires, is presented in Table 1. The majority of the participants lived in Vancouver, Canada (*n* = 103, 56%) with the remainder in Melbourne, Australia (*n* = 80, 44%). Over half the participants were in their 70s and approximately one-quarter were men. Twenty-one percent of participants reported at least 1 fall during the past year; not surprisingly, this is less than the anticipated rate of 35% of all adults > 65 years old [20] (our sample was limited to healthy community dwellers, and included younger participants age 60–64).

**Figure 2:**
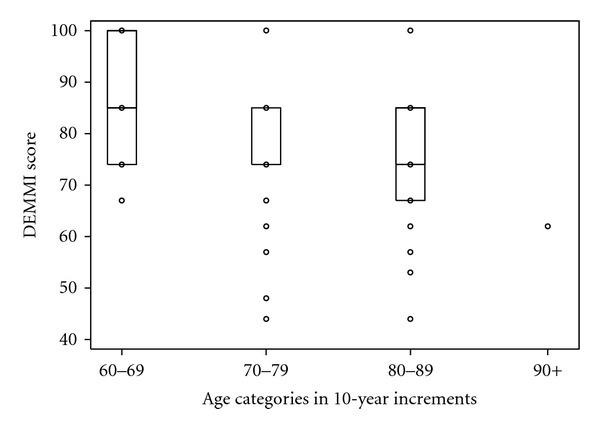
Box plot of DEMMI scores by age category with interquartile ranges (nb in age 70–79, median is the same as the 75th percentile; also, the oldest age category has only one observation).

**Figure 3:**
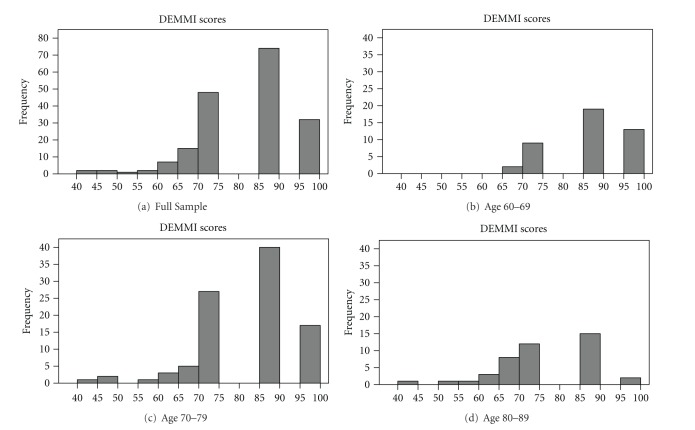
Histogram showing DEMMI scores for (a) total sample and (b) through (d) by age category (note category 90+ contained only 1 participant, therefore not represented graphically.

DEMMI scores are presented visually by age categories in 10-year increments (Figures 2 and 3). Normative data are provided for comparing group means (Table 2) as well as for comparing scores for individuals (Table 3). The second column of each table provides overall group scores. The next column labeled “independent” provides scores for those living fully independently in the community. Scores for men and women living independently are provided separately in the adjacent columns (though sex was not a statistically significant variable in this analysis, *P* = 0.49). Finally, a column labeled “semi-independent” includes men and women living in AL.

The mean DEMMI scores (Table 2) demonstrate a significant difference between age categories, with a lower score for each older age category (*P* values presented in Table 4). Reference intervals were presented in centiles (Table 3). The median score was maintained across each column with the exception of a lower median score for semi-independent in the 70–79 age category as well as semi-independent overall. Our finding that the 95th percentile was consistently high, with the exception of the semi-independent dwellers (and the overall sample which includes semi-independent dwellers), reflects the fact that 17.5% of the study sample scored the maximum of 100.

A priori, we were interested in comparing the DEMMI scores as a function of key variables including age category, sex, living situation, use of a mobility aid, country of origin, and falls history. For these categorical variables, we used Welch's two sampled *t*-tests to account for unequal variance between groups. Unadjusted *P* values (Table 4) showed statistical significance for age category, country, living situation, and mobility aid. Further exploration revealed that only 3 participants using mobility aids did not live in AL and only 4 participants using mobility aids lived in Vancouver (1 of whom also lived in AL). In addition, we noted the difference in DEMMI score by country (mean score in Canada 85.1 versus Australia 75.8, *P* = 0.00) was explained in part by the significant age difference by country (Australia 76.8 versus Canada 72.8, *P* = 0.00), and also by the proportion of individuals who resided in AL (Australia, 68.4% versus Canada, 7.8%, *P* = 0.00). The living situation could also be partly explained by age difference (AL 77.2 versus independent 73.1, *P* = 0.00). DEMMI scores did not differ by sex (*P* = 0.49) or reported falls history (*P* = 0.21).

## 4. Discussion

This study generates normative data for the DEMMI mobility instrument for adults over 60 years old who live independently or semi-independently in the community. We found that DEMMI mean scores generally decreased across increasing age categories, with the exception of independent-living men, whose average scores were highest in the 70–79 age category. Median scores on the other hand were generally maintained in the 60–69 and 70–79 age categories, with the exception of semi-independent 70–79-year olds, whose median score dropped to 74. Ninety-five percent of older adults living independently in the community demonstrated DEMMI scores ≥67, and 95% of those living semi-independently scored ≥48. Scores were as low as 44, though it was more likely that these individuals were using supports such as a mobility aid or living in AL. Overall, 17.5% of this sample scored the maximum possible of 100.

These normative data can be used either for comparing aggregate data or for comparisons of an individual. For example, in public health, one may wish to compare populations within a specific region (e.g., by neighbourhood or socioeconomic class) to inform policy makers regarding community accessibility, program evaluation, or city planning. In this case, the sample mean for a hypothetical population of fully independent community dwellers is 81.6. Looking at the bottom row of column 3 (“Independent”) in Table 2, this DEMMI mean score is below the lower limit of the 95% confidence interval of 82.5, important evidence that this region's older adult population overall demonstrates lower mobility compared to a healthy peer reference group.

More commonly, normative data is referred to when a clinician wishes to know how their patient compares to age- and sex-matched peers. For example, a family member may bring a 78-year-old female relative for evaluation as a result of concerns about increasing difficulties with activities of daily living such as managing her grocery shopping or maintaining her home. The clinician would obtain a DEMMI score during the initial assessment, then compare the patient's score of 62 to the reference intervals in row 3 (“Age 70–79”), column 4 (“Independent Women”) in Table 3 to see that a comparable healthy independent community-dwelling woman would score a median of 85, with 95 percent of the population scoring above 67. The clinician would then be able to use the results of this assessment to engage in meaningful education and goal setting with the patient and her family. Over the course of treatment, the DEMMI would be periodically re-administered to evaluate the patient's response to treatment and to guide ongoing treatment planning. An end goal for treatment could be set in consultation with the patient that either targeted a certain score (e.g., 74) or identified an item the patient would like to achieve (e.g., pick a pen up off the floor).

In a previous study, De Morton et al. [13] revealed that older adults with acute medical hospital admissions were discharged home with mean DEMMI scores of 60. The current study reveals higher mean scores than this, for community dwellers aged 60+ (Table 2). However, it would be expected that those recovering from acute illness and hospitalization would very likely demonstrate lower mobility at the time of discharge; mobility would be expected to improve with ongoing recovery and rehabilitation (“rehab potential” is an important factor in discharge planning). Therefore, the current study supports previous findings and demonstrates that means will likely differ between healthy community dwellers compared to community dwellers recovering from acute illness. The reference data obtained in this study could be used in the hospital setting to facilitate physiotherapy discharge planning and to support decision making for funding expenditure for inpatient and community-based rehabilitation services after acute hospital discharge.

We note limitations with this study. First, this was a relatively small sample (*N* = 183), just 25% of whom were men. Previous studies have developed normative data for health instruments from sample populations as small as 32 [21], though most commonly have over 100 participants [22, 23]. Nonetheless, a larger sample would provide greater representation across all age groups (especially those 80–89 and 90+, given the expected shift in population demographics in the coming years) and therefore better confidence in the reference value estimates reported in this study. Further, our sample was one of convenience, and therefore brings into question how well our sample represents the population of interest. Finally, the current study demonstrated 17.5% of community-dwelling older adults scored the maximum possible score of 100, which raises concerns of a ceiling effect. McHorney and Tarlov suggest that up to 15% of participants scoring at either scale extreme is acceptable [24]; and Barber-Westin et al. report up to one third of participants scoring the highest or lowest-possible scores is acceptable [25]. Recognizing this, the DEMMI was designed to measure across a broad spectrum of abilities (acutely hospitalized to healthy community dwellers) with a targeted use for clinical settings, where documenting improvements among those with excellent health is not a primary goal [26]. During the development of the DEMMI, one of the hardest items (standing on one leg with eyes closed) [11] was removed as there were no participants who could complete this item in an acutely hospitalized older population, and this item hence negatively impacted on some of the clinimetric properties of the DEMMI. The present study does not provide convincing evidence to suggest that the inclusion of this item is warranted.

A strength of this study is its important contribution of reference data to the DEMMI mobility instrument, critical in its broad application to older adults across the mobility spectrum from acutely ill, and hospitalized to healthy community dwellers. Additionally, gathering data from ten different community sites in two different countries provide broader generalizability to the reference intervals provided in this study.

## 5. Conclusions

This study is the first to report normative data for the DEMMI and provides important reference data on community dwelling older adults for clinicians and researchers. Normative data for this instrument will improve the interpretability of DEMMI scores, giving instrument users the ability to compare individual or group scores with known scores of an age- and sex-matched population. Its applicability is broad and can be used for population health applications; annual screening for early signs of mobility decline; therapeutic goal setting; evaluation of changes in mobility during recovery; discharge planning. As the focus of healthcare continues to shift from acute to community care and preventative health approaches, the DEMMI will assist in enhancing the continuity of patient care across clinical settings.

## Figures and Tables

**Table 1:** Description of study participants (*N* = 183).

	*n*	%
Vancouver, Canada	103	56
Melbourne, Australia	80	44
Age mean (SD)	74.6 (6.7)	
60–69	43	23
70–79	96	52
80–89	43	23
90+	1	1
Women	136	74
≥1 fall in past year	8	21
Used mobility aid	28 (4/24)*	15
Lived in house or apartment^#^	120 (95/25)*	66
Assisted living/retirement village^#^	62 (8/54)*	34
Lived alone	92	50

*n separated by location (Vancouver/Melbourne) to illustrate that participant living arrangements varied by country due to site selection during study design.

^#^Data incomplete for 1 participant, therefore *n* = 182.

**Table 2:** DEMMI normative Scores for group comparisons: mean, 95% confidence interval, and number in each subsample.

Age category	Overall	Independent	Independent women	Independent men	Semi-independent

	Mean				
	(95% CI)				
	Number				
	**86.4**	**86.2 **	**87.2**	**82.8**	**87.4**
**60–69**	(83.2, 89.6)	(82.9, 89.6)	(83.3, 91.1)	(75.4, 90.1)	(70.4, 100)
	*n* = 43	*n* = 38	*n* = 30	*n* = 8	*n* = 5

	**81.4**	**84.3 **	**83.4**	**88.4**	**77.2**
**70–79**	(78.9, 83.9)	(81.5, 87.1)	(80.2, 86.6)	(82.1, 94.6)	(72.9, 81.6)
	*n* = 96	*n* = 57	*n* = 47	*n* = 10	*n* = 39

	**75.3**	**77.9 **	**78.6**	**76.0**	**71.9**
**80–89**	(71.8, 78.9)	(73.3, 82.5)	(73.3, 83.8)	(63.10, 88.9)	(65.9, 77.9)
	*n* = 43	*n* = 24	*n* = 18	*n* = 6	*n* = 18

**90+**	**62**	**62**	**—**	**62**	**—**
	*n* = 1	*n* = 1		*n* = 1	

	81.0	83.5	83.7	82.6	76.5
Total	(79.3, 82.8)	(82.5, 85.4)	(81.4, 85.9)	(77.9, 87.2)	(73.1, 79.9)
	*n* = 183	*n* = 120	*n* = 95	*n* = 25	*n* = 62

**Table 3:** DEMMI reference intervals for individual comparisons, median (5th, 95th percentiles). Please refer to Table 2 for subsample sizes.

Age category	Overall	Independent	Independent women	Independent men	Semi-independent

	Median (p5, p95)				
**60–69**	**85** (74,100)	**85** (74, 100)	**85** (74,100)	**85 **(74,100)	**85 **(67, 100)
**70–79**	**85** (62,100)	**85 **(67, 100)	**85 **(67,100)	**85 **(74,100)	**74 **(48, 100)
**80–89**	**74** (57,85)	**74 **(62, 100)	**85**(57,100)	**74** (67,100)	**74 **(44, 85)
**90+**	62*	62*	**—**	62*	—

Total	85 (62,100)	85 (67, 100)	85 (67,100)	85 (67,100)	74 (48, 100)

*Only 1 observation.

**Table 4:** Unadjusted *P*-values from Welch's two sample *t*-tests comparing DEMMI scores amongst variables of interest.

Variable	*P*-value
Age 70–79 (versus 60–69)	0.02
Age 80–89 (versus 60–69)	0.00
Age 80–89 (versus 70–79)	0.01
Sex	0.49
Falls history	0.21
Independent versus semi-independent living	0.00
Country	0.00
Mobility aid use	0.00
